# Therapeutical Targets in Allergic Inflammation

**DOI:** 10.3390/biomedicines10112874

**Published:** 2022-11-09

**Authors:** Lorenzo Salvati, Francesco Liotta, Francesco Annunziato, Lorenzo Cosmi

**Affiliations:** 1 Department of Experimental and Clinical Medicine, University of Florence, 50134 Firenze, Italy; 2 Immunology and Cell Therapy Unit, Careggi University Hospital, 50134 Firenze, Italy; 3 Flow Cytometry Diagnostic Center and Immunotherapy (CDCI), Careggi University Hospital, 50134 Firenze, Italy

**Keywords:** allergy, asthma, atopic dermatitis, CRSwNP, desensitization, dupilumab, efficacy, eosinophilic esophagitis, immunotherapy, omalizumab, practical approach, safety, urticaria

## Abstract

From the discovery of IgE to the in-depth characterization of Th2 cells and ILC2, allergic inflammation has been extensively addressed to find potential therapeutical targets. To date, omalizumab, an anti-IgE monoclonal antibody, and dupilumab, an anti-IL-4 receptor α monoclonal antibody, represent two pillars of biologic therapy of allergic inflammation. Their increasing indications and long-term follow-up studies are shaping the many different faces of allergy. At the same time, their limitations are showing the intricate pathogenesis of allergic diseases.

## 1. Introduction

In recent decades, the treatment of many allergic diseases has been revolutionized by the use of biologic therapies, particularly in severe forms. Allergic inflammation has been extensively investigated to identify potential therapeutical targets. Among these, omalizumab and dupilumab constitute two biologic therapies whose mechanisms of action, indications, and adverse events must be known by physicians in order to make appropriate therapeutic decisions. As the on-label indications and off-label uses are increasing, specialists who prescribe these monoclonal antibodies should be able to identify those patients that are likely to respond to treatment, as well as being aware of serious adverse events. This narrative review will explore the premises and consequences of targeting allergic inflammation with omalizumab or dupilumab, comparing these two biologic therapies in different allergic diseases involving the respiratory tract (asthma and chronic rhinosinusitis with nasal polyps), the skin (chronic spontaneous urticaria and atopic dermatitis), and the gastrointestinal tract (eosinophilic esophagitis), as well as their potential role in allergy prevention, especially as adjuvants in immunotherapy or desensitization protocols. The most relevant studies of omalizumab and dupilumab for the treatment of allergic diseases have been selected by the authors from the literature. The aim of this narrative review is to summarize and discuss the most important known and emergent uses of omalizumab and dupilumab in targeting allergic inflammation, as well as current safety concerns.

## 2. Methods

### 2.1. Search Strategy and Selection Criteria

We searched the medical literature with no time restrictions on July 2022 using MEDLINE and Embase to identify pertinent articles with the medical subject heading terms “omalizumab” OR “dupilumab” OR “asthma” OR “atopic dermatitis” OR “chronic rhinosinusitis with nasal polyps” OR “desensitization” OR “eosinophilic esophagitis” OR “immunotherapy” OR “type 2 inflammation” OR “urticaria”. We included only publications published in English and selected those with findings that were, in our view, of the greatest importance, favouring randomised controlled trials, meta-analyses, systematic reviews, guidelines, consensus documents, and high-quality comprehensive reviews. We predominantly selected papers from the past 4 years, but also included highly regarded older publications. In addition, we included relevant publications identified via reference lists of articles that were collected by the search strategy or were otherwise identified by the authors. 

### 2.2. On-Label and Off-Label Definitions

“On-label” uses refer to those that are approved by regulatory pharmaceutical agencies (i.e., the Food and Drug Administration [FDA] or European Medicines Agency [EMA]). On the contrary, “off-label” uses refer to those that are unapproved (e.g., different indication, different patient age, different dose, different dosage form). In this narrative review, off-label refers to the use of omalizumab or dupilumab for a disease or medical condition that is not approved by regulatory pharmaceutical agencies to be treated with that biologic therapy.

## 3. Mechanisms of Action: Targeting Allergic Inflammation

Omalizumab is an IgG1κ humanized monoclonal antibody that binds free IgE, inhibiting the binding to high- and low-affinity IgE receptors (FcεRI and FcεRII, respectively), while dupilumab is an IgG4 human monoclonal antibody that binds the α subunit of the IL-4 receptor (IL-4Rα), one of the two subunits of the IL-4 and IL-13 receptors, respectively, thus inhibiting the signaling of both IL-4 and IL-13 [1,2,3,4]. Beyond the basic mechanisms of action, it is now known that both these monoclonal antibodies have pleiotropic effects in the control of allergic inflammation.

In detail, omalizumab binds free IgE at the Cε3 domains with a binding affinity higher than that observed between IgE and its receptor, and forms trimers and hexamers [5,6,7]. This prevents IgE from binding FcεRI on mast cells and basophils, limiting their activation and degranulation [7]. IgEs already bound to FcεRI are not targeted by omalizumab, thereby no cross-linking is mediated by this anti-IgE monoclonal antibody [8,9]. Significant reduction in basophil FcεRI expression occurs early within the first week of treatment, while a few months of therapy are needed to reduce FcεRI expression on mast cells [10]. Moreover, omalizumab is able to detach pre-bound IgE from its receptor on basophils and mast cells [11,12,13]. Long-term treatment is associated with reduced CD40L (CD154) expression on circulating Th cells and lower frequencies of plasmacytoid dendritic cells (pDC) [11]. Reduction of eosinophils in terms of peripheral counts and tissue infiltration can be an indirect effect of omalizumab [14,15].

On the other hand, dupilumab inhibits the signaling of both IL-4 and IL-13 which are two key effector cytokines in allergic inflammation. Type I (IL-4Rα/γc) and type II (IL-4Rα/IL-13Rα) IL-4 receptors are widely expressed by hematopoietic cells (e.g., B cells, Th cells, eosinophils), epithelial cells, and airway smooth-muscle cells [16,17]. These cytokines induce the differentiation of Th2 lymphocytes and the activation and class switching of B cells towards the production of IgE, and promote the recruitment of eosinophils, both directly by increasing the expression of adhesion molecules and indirectly via chemokines (such as eotaxin) produced by epithelial cells. Furthermore, IL-13 induces the expression of the nitric oxide synthase (iNOS) by epithelial cells, promotes mucus production and goblet cell hyperplasia, stimulates the contraction and proliferation of bronchial smooth-muscle cells and also increases the extracellular deposition of collagen and fibroblasts, thus promoting airway remodeling [18,19]. IL-13 also has relevant functions at the skin level [20]. 

## 4. Severe Asthma

### 4.1. Omalizumab vs. Dupilumab in Severe Asthma

Omalizumab is indicated in the treatment of severe persistent allergic asthma, while dupilumab is indicated in the treatment of severe type 2 asthma [1,21,22]. These definitions include phenotypes of asthma that are very often overlapping [23]. For omalizumab, sensitization to a perennial aeroallergen is a prerequisite for prescription in patients with asthma [24].

A systematic review of the European Academy of Allergy and Clinical Immunology (EAACI) has compared omalizumab to standard of care for severe allergic asthma [25]. It has demonstrated high certainty of the evidence (Grading of Recommendations Assessment, Development, and Evaluation, GRADE) in relation to the reduction of the annual rate of clinically significant asthma exacerbations (assessed with annualized rate) [25,26,27,28,29,30,31], decrease of inhaled corticosteroids (ICS) and rescue medication use [26,32,33,34,35,36,37], followed by moderate certainty of the evidence in relation to increased asthma control (assessed with ACQ-6) [27,37,38] and reduced fractional exhaled nitric oxide (FeNO) [28,34,36], but low certainty of the evidence in relation to the improvement of FEV1 [26,30,39,40,41,42].

Similarly, dupilumab, compared to standard of care, has been investigated in the same setting [25], showing high certainty of the evidence (GRADE) in relation to the reduction of clinically significant exacerbation rate ratio (assessed with annual asthma exacerbations) and improved asthma control (assessed with ACQ-5), while low when considering FEV1 amelioration [43].

For omalizumab (approved for asthma in 2003), real-life studies reach up to 16 years and show maintenance of both clinical (reduced exacerbations, improved asthma control) as well as functional response in patients with moderate–severe asthma [44,45]. For dupilumab (approved for asthma in 2018), reduction of the annual exacerbation rate and functional improvement is maintained for up to 96 weeks as shown in the open-label extension TRAVERSE study [46].

Omalizumab, in case of clinical need, can be administered during pregnancy [47]. Among pregnant women exposed to omalizumab, the EXPECT study did not show an increased risk of major congenital anomalies or thrombocytopenia in the newborns [48,49]. A trial is ongoing in women undergoing fertility treatment with the primary outcome of efficacy of omalizumab in increasing pregnancy rate in females with asthma compared to placebo [50]. On the contrary, considering dupilumab, the Pregnancy Exposure Study, a post-authorization safety study to monitor pregnancy and infant outcomes following administration of dupilumab during planned or unexpected pregnancy, is ongoing [51]. While through the placenta IgG1 are preferentially transported to the fetus, followed by IgG4, IgG3, and IgG2, particularly during the third trimester of pregnancy, overall secretion of IgG is scarce in breast milk [47,52,53].

In children aged between 6 and 11 years, the comparison between omalizumab and dupilumab becomes interesting. The first study of omalizumab in this age group dates back to 2001, showing efficacy in the control of symptoms, reduction of exacerbations and steroid-sparing effect [33]. Starting from the observations in the control of children’s asthma, the potential role of omalizumab in the anti-viral response has been hypothesized and it has been found that omalizumab enhances the production of IFN-α by pDC, thus reducing viral-induced exacerbations [54]. Recently, the VOYAGE study showed a reduction in the rate of exacerbations, improved symptom control and lung function in children treated with dupilumab [55].

To date, there are no head-to-head clinical trials that compare omalizumab and dupilumab in patients with severe asthma. A recent indirect comparison investigated the efficacy and safety of dupilumab vs. omalizumab in severe allergic asthma after 20–32 weeks and after 48–52 weeks of treatment [56]. The results showed no differences in the exacerbation rate (assessed as mean reduction of annual exacerbations and percentage of patients without exacerbations), asthma control (assessed with ACQ) and quality of life (assessed with AQLQ) [45]. Only lung function showed significant results with an absolute mean difference in FEV1 of +96 mL (11–182 mL) at 48–52 weeks of treatment in favor of dupilumab, but the improvement was below the prespecified minimal clinically important difference (200 mL) [45]. Notably, this indirect comparison has some risk of bias due to incomplete descriptions in the included studies and the general quality of evidence assessed by GRADE being considered very low, due to low comparability of the studies [56].

In patients with severe type 2 asthma, eligible for add-on type 2-targeted biologics, the choice of anti-IgE or anti-IL-4Rα strategy can be challenging [1,24,57]. Blood eosinophil count, fractional exhaled nitric oxide (FeNO), allergen-driven symptoms and time of onset of asthma can be used to guide the choice of biologics, but have many limitations [24,58,59]. Total IgE levels are used to define the dosage of omalizumab in addition to body weight [60]. Although additional effects resulting from the contemporary blocking of IgE and inhibition of IL-4 and IL-13 signaling might be expected, there are no indications of the combined use yet. Finally, there are clinical scenarios in which the shift from omalizumab to dupilumab or vice versa can be taken into consideration, particularly in those cases where there is a lack of response to one or the other monoclonal antibody. In such conditions, it is essential to reconsider differential diagnosis, reassess patient adherence to therapy, investigate coexisting conditions, and redefine asthma phenotype by integrating biomarkers into the clinical and functional evaluation before starting a new biologic, both in adults and in children [61,62]. 

### 4.2. Other Biologics in Severe Asthma

Many other biologics have been approved for the treatment of severe type 2 asthma [1,57,63]. Mepolizumab and reslizumab are anti-IL-5 monoclonal antibodies that prevent IL-5 from binding to IL-5Rα, hence inhibiting the maturation, activation, proliferation, and recruitment of eosinophils in the airways [63,64,65,66,67,68]. Benralizumab binds the α subunit of the IL-5R (IL-5Rα), and its afucosylated site augments the binding to FcγRIIIa, determining antibody-dependent cell-mediated cytotoxicity (ADCC) by NK cells [69,70,71]. Recently, the FDA has approved tezepelumab as maintenance therapy for severe asthma. This is the first approved monoclonal antibody targeting thymic stromal lymphopoietin (TSLP), an epithelial cytokine released by the airway epithelium that acts as early mediator at the top of the allergic inflammatory cascade in asthma [72,73,74].

## 5. Chronic Rhinosinusitis with Nasal Polyps

Omalizumab and dupilumab are both approved for the treatment of severe chronic rhinosinusitis with nasal polyps (CRSwNP) [75,76]. CRSwNP is a frequent comorbidity in asthmatics and it is present in around 40% of patients with severe asthma [77,78]. Monoclonal antibodies are changing the traditional approach to patients with CRSwNP [79]. In fact, allergic inflammation has a central role in disease pathogenesis and type 2 inflammation represents one of the major CRSwNP endotypes [80]. It has been shown that in these subjects, nasal bacteria-reactive B cells differentiate into IgE-producing B cells, contributing to CRSwNP pathogenesis [81]. 

Omalizumab improved endoscopic, clinical, and patient-reported outcomes in severe CRSwNP with inadequate response to intranasal corticosteroids at 24 weeks of treatment, with further modest decrease in polyp and congestion scores from week 24 to week 52 [82,83,84]. When omalizumab was suspended, gradual increases in polyp and congestion scores were observed by week 76, without reaching pretreatment levels [76]. This observation suggests a prolonged rather than a permanent disease-modifying effect [84]. 

Dupilumab has been demonstrated to reduce polyp size, sinus opacification, and severity of symptoms in patients with severe CRSwNP regardless of eosinophilic status [85,86,87]. Indirect comparison analyses based on clinical trials data showed that dupilumab has the most beneficial effects in improving symptoms, sense of smell and health-related quality of life, in reducing rescue oral corticosteroids and rescue nasal polyp surgery, as well as in decreasing nasal polyp size and nasal congestion severity [88,89,90].

Biologics are reshaping the management of CRSwNP by demonstrating that a multidisciplinary approach—also integrating histopathologic data—is essential in deciding the best approach for a patient with CRSwNP [75,79,91].

## 6. Urticaria and Atopic Dermatitis

Omalizumab has changed the management of chronic spontaneous urticaria (CSU) and to date represents the treatment of choice in patients with CSU unresponsive to second-generation H1-antihistamines [92]. Omalizumab controls both signs and symptoms of CSU, reducing wheals and itch and improving the quality of life of affected patients [93,94,95,96,97]. Retreatment is safe and clinically effective: as the number of retreatments increases, the percentage of patients achieving complete remission of CSU increases, and time to complete clinical response reduces [98,99]. Biosimilars are now in development for CSU, such as CT-P39 [100]. In addition, ligelizumab has been developed. Ligelizumab is a second-generation anti-IgE monoclonal antibody with a higher affinity for IgE and slower offloading time compared to omalizumab [101,102]. Ligelizumab showed superiority compared with placebo, but not omalizumab [103].

Dupilumab has revolutionized the therapy of atopic dermatitis (AD), representing the first monoclonal antibody approved for the treatment of moderate-to-severe AD of adults (in 2016 FDA, 2017 EMA), adolescents aged 12 to 18 years (in 2019 FDA and EMA), children aged 6 to 11 years (in 2020 FDA and EMA) and even children aged 6 months to 5 years (in 2022 FDA), whose eczema is not adequately controlled by topical therapies, or when those therapies are not advisable [104,105,106,107,108]. Type 2 inflammation is a major endotype in AD [108,109]. The recent approval in children aged 6 months to 5 years provides for the first time a systemic therapeutic option in this population with unprecedented expectations [110].

## 7. Eosinophilic Esophagitis

Recently, dupilumab has been approved by the FDA for the treatment of adults and children aged 12 and older (who weigh at least 40 kg) with eosinophilic esophagitis [111,112,113]. Dupilumab treatment resulted in a significant symptomatic, endoscopic, and histologic improvement in patients with eosinophilic esophagitis [112,114,115]. Allergic inflammation is crucial in the recruitment of eosinophils to the esophagus; in fact, IL-13 is over-expressed in biopsy specimens obtained from these patients [116,117]. Similarly to other atopic diseases, different endotypes have been identified in eosinophilic esophagitis [118,119,120]. 

## 8. Prevention of Allergy

The potential role of anti-IgE and anti-IL-4Rα therapeutic strategies in the management of patients undergoing immunotherapy or desensitization is under discussion, and it is of great interest considering their adjuvant function in preventing severe Type I hypersensitivity reactions. 

In 2006, Casale et al. showed that pretreatment with omalizumab significantly decreased acute allergic reactions after rush immunotherapy (RIT) for ragweed-induced seasonal allergic rhinitis [121]. Patients under immunotherapy receiving omalizumab had a fivefold reduction in risk of anaphylaxis caused by RIT [121]. In response, Matheu et al. presented the case of a 27-year-old man with type I diabetes mellitus and insulin allergy who, after desensitization, still had allergic symptoms that fully abated with omalizumab treatment allowing insulin re-administration [122]. Omalizumab has been administered in allergen immunotherapy, oral food desensitization, venom immunotherapy, and before aspirin desensitization in management of patients with aspirin-exacerbated respiratory disease contributing to prevent reactions [123,124,125,126,127,128]. Omalizumab has also been successfully used as adjuvant treatment in desensitization to chemotherapeutic drugs (i.e., carboplatin and oxaliplatin) in those patients experiencing breakthrough IgE-mediated reactions during desensitization protocol [129,130,131,132,133,134,135]. It has also been used to prevent hypersensitivity reactions to monoclonal antibodies (infliximab and rituximab) during desensitization [136,137]. Omalizumab could help reduce the impact of breakthrough reactions or even prevent reactions during desensitization by depowering mast cells and basophils. 

In adults with grass-pollen seasonal allergic rhinitis, dupilumab administered in combination with subcutaneous immunotherapy (SCIT) significantly reduced the number of systemic reactions to SCIT [138]. In 2019, a patient under dupilumab treatment for atopic dermatitis developed tolerance to foods that previously induced an allergic reaction [139]. There are now several ongoing clinical trials to investigate dupilumab as either a monotherapy or as an adjuvant to oral immunotherapy for patients with peanut allergy [140]. Dupilumab might contribute to prevent reactions during allergen-specific immunotherapy by controlling T2 inflammation.

## 9. Safety Concerns

Injection site reactions are the most common adverse reaction for both omalizumab and dupilumab. Since the first approval of omalizumab in asthma was 14 years earlier than that of dupilumab in atopic dermatitis, there are longer-term real-life safety data for omalizumab compared to dupilumab. 

Over time, the incidence of anaphylaxis associated with omalizumab administration has been a rare event [141,142]. Notably, in 2007 the FDA issued a boxed warning about the risk of omalizumab-associated anaphylaxis, but the incidence of anaphylaxis in different datasets was highly variable [142,143,144,145]. Similarly, serum sickness is infrequent, but it should be suspected in patients recently treated with omalizumab (3–10 days) that present with constitutional symptoms, fever, lymphadenopathy, and arthralgia [142,146,147,148]. 

A relatively common adverse event during treatment with dupilumab for atopic dermatitis is conjunctivitis, which in contrast appears to be rare in patients treated for severe asthma [149,150,151,152]. Dupilumab-induced conjunctivitis generally responds well to topical steroids with or without topical cyclosporine [149,153]. 

Patients treated with dupilumab can experience transient increases in blood eosinophil counts, but in some patients, dupilumab-induced eosinophilia can become severe [154,155]. Dupilumab for the treatment of asthma is not suggested if blood eosinophils (current or historic) are greater than 1500 cells per μL [61,156]. 

Owing to the abrupt discontinuation of oral corticosteroids and/or misdiagnosis, the development of eosinophilic granulomatosis with polyangiitis (EGPA) after initiation of treatment with omalizumab or dupilumab has been reported [157,158,159,160]. The association of adult-onset asthma and CRS should be an alert regarding potential underlying vasculitis [161]. For this reason, patients must always be accurately evaluated before starting therapy and in the course of omalizumab or dupilumab treatment. Dupilumab has also been associated with the occurrence of chronic eosinophilic pneumonia [162]. 

The risk of parasitic infections is debated. Patients at high risk of helminth intestinal infections treated with omalizumab showed a modest increase in the incidence of infection, but disease severity and response to anthelmintics were both unaffected by concomitant omalizumab therapy [163,164]. Considering dupilumab, pooled analyses of clinical trials data showed no increased risk of helminthic infections between the placebo and dupilumab treatment groups both in children and in adults, but studies were conducted mostly in North America and Europe and did not include endemic areas for parasites [165,166].

## 10. Conclusions and Perspectives

**Figure 1:**
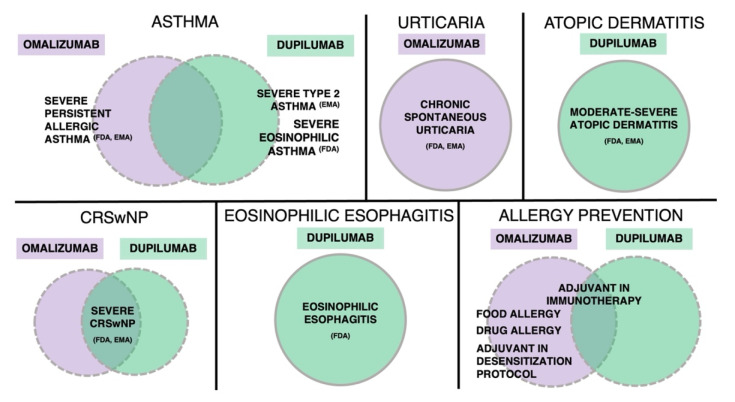
On-label indications and off-label promising uses of omalizumab and dupilumab in allergic inflammation (as of October 2022). Omalizumab is approved as an add-on treatment in severe persistent allergic asthma (FDA, EMA), similarly to dupilumab in severe type 2 asthma (EMA) and asthma with eosinophilic phenotype (FDA) or oral corticosteroid-dependent asthma, regardless of phenotype (FDA). Regarding allergic skin diseases, omalizumab is approved for the treatment of chronic spontaneous urticaria (FDA, EMA), similarly to dupilumab for the treatment of moderate-severe atopic dermatitis (FDA, EMA). Both biologics have been approved in patients with severe chronic rhinosinusitis with nasal polyps (FDA, EMA). In eosinophilic esophagitis, dupilumab has recently been approved by the FDA. Considering prevention of allergic reactions, both biologics are under investigation as adjuvant in oral immunotherapy. Omalizumab is used off-label as adjuvant in desensitization protocols to prevent breakthrough reactions. FDA denotes U.S. Food and Drug Administration, EMA European Medicines Agency.

In conclusion, the choice of omalizumab vs. dupilumab in allergic inflammation must be based on an extensive phenotypic and endotypic characterization of the patient with allergic disease. In this narrative review, we considered the most relevant on-label and off-label uses of these two biologics targeting allergic inflammation (Figure 1). Accurate predictive and monitoring biomarkers are needed to inform treatment strategies. Head-to-head studies of monoclonal antibodies in patients with allergic diseases are expected in the near future, which may not be so far, considering for instance the EVEREST (EValuating trEatment RESponses of Dupilumab Versus Omalizumab in Type 2 Patients) trial, which is enrolling patients with CRSwNP and coexisting asthma to evaluate treatment responses of omalizumab vs. dupilumab [167,168].

## Data Availability

Not applicable.
